# NLRP3 Inflammasome at the Interface of Inflammation, Endothelial Dysfunction, and Type 2 Diabetes

**DOI:** 10.3390/cells10020314

**Published:** 2021-02-03

**Authors:** Ilona M. Gora, Anna Ciechanowska, Piotr Ladyzynski

**Affiliations:** Nalecz Institute of Biocybernetics and Biomedical Engineering, Polish Academy of Sciences, Ks. Trojdena 4, 02-109 Warsaw, Poland; aciechanowska@ibib.waw.pl (A.C.); pladyzynski@ibib.waw.pl (P.L.)

**Keywords:** NLRP3 inflammasome, inflammation, type 2 diabetes mellitus, endothelial cells

## Abstract

Type 2 diabetes mellitus (T2DM), accounting for 90–95% cases of diabetes, is characterized by chronic inflammation. The mechanisms that control inflammation activation in T2DM are largely unexplored. Inflammasomes represent significant sensors mediating innate immune responses. The aim of this work is to present a review of links between the NLRP3 inflammasome, endothelial dysfunction, and T2DM. The NLRP3 inflammasome activates caspase-1, which leads to the maturation of pro-inflammatory cytokines interleukin 1β and interleukin 18. In this review, we characterize the structure and functions of NLRP3 inflammasome as well as the most important mechanisms and molecules engaged in its activation. We present evidence of the importance of the endothelial dysfunction as the first key step to activating the inflammasome, which suggests that suppressing the NLRP3 inflammasome could be a new approach in depletion hyperglycemic toxicity and in averting the onset of vascular complications in T2DM. We also demonstrate reports showing that the expression of a few microRNAs that are also known to be involved in either NLRP3 inflammasome activation or endothelial dysfunction is deregulated in T2DM. Collectively, this evidence suggests that T2DM is an inflammatory disease stimulated by pro-inflammatory cytokines. Finally, studies revealing the role of glucose concentration in the activation of NLRP3 inflammasome are analyzed. The more that is known about inflammasomes, the higher the chances to create new, effective therapies for patients suffering from inflammatory diseases. This may offer potential novel therapeutic perspectives in T2DM prevention and treatment.

## 1. Introduction

In 2019, the International Diabetes Federation (IDF) estimated that 463 million people aged 20 to 79 years had diabetes. This represents 9.3% of the world’s population in this age group. It is projected that this number will reach 578 million by 2030 and 700 million by 2045 [1]. According to the IDF estimates, the number of deaths that were attributed to diabetes and complications of diabetes in 2019 was 4.2 million. The annual global health expenditure on diabetes is estimated to be 760 billion US dollars, and this number is projected to reach 845 billion US dollars by 2045. The above data prove that diabetes is one of the most common diseases and major public health burdens worldwide [2,3,4,5].

Type 2 diabetes mellitus (T2DM) accounts for 90% to 95% of all diabetes in the world, which makes it the most common type of diabetes, with a prevalence reaching 8.3% in 2014 [2]. The T2DM is characterized by insulin resistance, which leads to hyperglycemia and, consequently, to hyperinsulinemia in order to lower the rise in blood glucose levels. Gradually, a relative inadequate production of insulin may develop as a result of pancreatic islet β-cell dysfunction. It is more common for T2DM to be detected in older adults, although the prevalence of the disease in children and adolescents is continuously rising due to the growing levels of obesity, sedentary lifestyle, and poor dietary habits, including excessive consumption of red meat, processed food, and sweet beverages.

A number of studies have clearly demonstrated that chronic tissue inflammation is a key contributing factor to T2DM [6,7]. It has been shown that elevated levels of inflammatory proteins such as interleukins (IL) 1β and 6, monocyte chemoattractant protein 1 (MCP1), and C-reactive protein (CRP) are predictive of T2DM [8]. Inflammation is the first response of the immune system to infection or tissue injury and is a significant component in the pathogenesis of many vascular disorders such as atherosclerosis and diabetic angiopathy [9,10].

Pivotal research into the nucleotide-binding oligomerization domain-like receptors (NOD), leucine-rich repeats (LRR), and pyrin domain-containing protein 3 (NLRP3) inflammasome complex suggests that it acts as a trigger initiating inflammatory responses, playing a crucial role in the dysfunction of endothelial cells. The NLRP3 inflammasome also makes a significant contribution during the development of diabetes and its complications [11].

The aim of this work is to present a review of the links between the NLRP3 inflammasome, endothelial dysfunction, and type 2 diabetes.

## 2. The NLRP3 Inflammasome and Inflammatory Regulators

### 2.1. NLRP3 Inflammasome

Inflammasomes are large intracellular multi-protein complexes with a molecular mass of at least 700 kDa, containing caspase-1, cysteine-aspartic proteases 1 apoptosis-associated speck-like protein (ASC), and nucleotide-binding oligomerization domain-like receptor with a pyrin domain (NLRP). This complex is present in the cytosol of stimulated immune cells such as monocytes, macrophages, and dendritic cells [12,13,14,15].

Two types of inflammasomes have been recognized so far: Nod-like receptor (NLR) inflammasomes, and Pyrin and HIN domain-containing protein (PYHIN) inflammasomes. The NLRs are intracellular receptors of the immune system, and a subset of them including NLRP1, NLRP2, NLRP3, NLRC4, NLRP6, NLRP7, and NLRP12 acts as a component of inflammasome complexes with sensory roles. The PYHIN inflammasomes act as sensory parts and can interact with viral or bacterial double-stranded DNA (dsDNA) [16,17]. The inflammasome plays an important role in the pathogenesis of various human inflammatory diseases [18,19]. The concept of inflammasome was proposed for the first time by the Tschopp research group in 2002 [20].

The NLRP3 inflammasome has been the most fully characterized so far. The NLRP3 contains three domains: C-terminal leucine-rich repeats (LRRs), a central nucleotide domain termed as the NACHT domain, and an N-terminal effector domain, i.e., pyrin domain (PYD). The ASC contains an N-terminal PYD and a C-terminal caspase recruitment domain (CARD). Caspase-1 contains CARD and catalytic domains [21,22,23,24]. The NLRP3 inflammasome is expressed in the cytosol of various cell types, including granulocytes, monocytes, macrophages, dendritic cells, T and B cells, epithelial cells, osteoblasts, fibroblasts, and melanoma cells [25,26].

The NLRP3 Pattern Recognition Receptors (PRRs) are able to recognize molecules identifying pathogens—the so-called Pathogen-Associated Molecular Patterns (PAMPs), or molecules released by damaged cells, i.e., the Damage-Associated Molecular Patterns (DAMPs). The PRRs are divided into the following families: the toll-like receptors (TLRs), the nucleotide binding and oligomerization domain (NOD)-like receptors (NLRs), the retinoic acid inducible gene-I (RIG-I)-like receptors (RLRs), the absentin-melanoma (AIM)-like receptors (ALRs), and the C-type lectins (CTLs) [27,28].

### 2.2. Caspase-1

Caspases are cysteine proteases. Their primary role is to regulate apoptosis. However, there is evidence available that the primary activity of some of caspases controls inflammation [29,30]. Pro-inflammatory caspases are known as group I caspases. Thirteen caspases have been identified in mammals. Four of them, i.e., caspase-1, -4, -5, and -12, play a role of pro-inflammatory caspases in humans, whereas in mice, there are three such caspases, i.e., -1, -11, and -12. Out of all the pro-inflammatory caspases, caspase-1 has been studies and characterized in the most detail [31,32].

The crucial role of caspase-1, known as interleukin-1β-converting enzyme (ICE), in inflammation is to catalyze the intracellular conversion of the pro-inflammatory cytokines pro-IL-1β (31 kDa) and pro-IL-18 (24 kDa) into their mature and biologically active forms IL-1β (17.5 kDa) and IL-18 (18 kDa), respectively [33,34]. This step is a prerequisite to releasing cytokines from the cytosol into the extracellular space, which in turn allows them to exert their pro-inflammatory impact on the receptors of surrounding cells in a paracrine manner [35]. Proteases such as elastase, cathepsin G, and proteinase 3 are able to activate IL-1β independently of caspase-1 [36]. Caspases are synthesized as zymogens. One of the important functions of inflammasomes is to mediate the cleavage of the caspases, which activates them [37].

### 2.3. Interleukin 1β

IL-1 is a master regulatory cytokine. This inflammatory/immune mediator plays an important role at a few levels of the immune responses, e.g., it activates cells to produce other inflammatory cytokines and chemokines [38].

The IL-1β is a potent pro-inflammatory cytokine. IL-1β induces endothelial cells to express cell membrane adhesion molecules and pro-coagulant activity. Hence, trapping leukocytes from the bloodstream is possible [39]. When PRR proteins of the NLRP3 inflammasome recognize molecules identifying PAMPs or DAMPs, then IL-1β is transcriptionally regulated [40,41,42].

Two signals are required for the production of IL-1β. The first signal is the synthesis of intracellular pro-IL-1β, which occurs after the stimulation of PRRs, such as TLRs situated in endosomes or on the cell surface and NLRs located in the cytoplasm, i.e., the ligation of PRR with its respected ligand. The second signal is the recruitment and activation of pro-caspase-1 and subsequent processing (proteolytic cleavage) of pro-IL-1β by caspase-1 to its mature form, i.e., IL-1β [43,44]. In most cases, the recruitment of pro-caspase-1 is facilitated by ASC.

The IL-1β is not only secreted by immune cells such as monocytes or macrophages, neutrophils, lymphocytes B, dendritic cells, and NK cells but also by nonimmune cells such as keratinocytes [45]. The potent pro-inflammatory cytokine IL-1β is known to contribute to the inflammatory response in various metabolic diseases including diabetes [46,47]. However, the mechanism by which IL-1β is induced in a distinctive metabolic dysfunction has only recently come to light and seems to be connected with inflammasome platform activation [48,49].

In 2011, Menu and Vince recapitulated the role of abnormal activation of NLRP3 inflammasome and the overexpression of IL-1β in the development of diabetes and in its complications [50]. Clinical studies suggest that IL-1β is linked to adipocyte inflammation and insulin resistance and that the factors controlling bioactive IL-1β secretion have therapeutic implications [51,52]. IL-1β decreases tyrosine phosphorylation of insulin receptor substrate-1 (IRS-1) and its gene expression. This inhibits the downstream pathway of insulin signaling, which is required to control glycemia, thereby inducing insulin resistance [53]. Moreover, IL-1β contributes to the expression of tumor necrosis factor alpha (TNFα), which also promotes insulin resistance. The insulin sensitivity may be reduced even further because of the overaccumulation of lymphocytes in adipose tissues, which is mediated by IL-1β and IL-18 [54]. In mice fed a high-fat diet (HFD), the lack of IL-1β was protective in terms of adipose tissue inflammation and insulin resistance [55,56].

IL-1β cytokine works in two planes. On the one hand, IL-1β interferes with insulin signaling in hepatocytes and adipocytes, inhibits insulin-induced glucose uptake, suppresses lipogenesis, and decreases the release of adiponectin [57]. On the other hand, IL-1β has been reported to contribute to β-cell failure. High levels of IL-1β, induced by high levels of glucose or free fatty acids, contribute to decreased insulin secretion, impaired β-cell replication, and increased β-cell apoptosis, whereas low levels of IL-1β have the opposite effects in pancreatic islets [58]. Based on clinical observations, IL-1β has been shown to reduce the insulin secretion and a number of β cells [59]. IL-1β influences the activation of mitogen-activated protein kinases (MAPK) and the nuclear factor kappa-light-chain-enhancer of activated B cells (NF-κB), which in turn control the expression of the encoding genes in the process of β-cell death [60]. Presumably, in the pre-T2DM stage, insulin resistance reduces glucose uptake, causing transient postprandial hyperglycemia. However, glucose-induced IL-1β production and NF-κB activation are unlikely to be the main mediators of β-cell glucotoxicity, and transient hyperglycemia is unlikely to be sufficient to trigger the transition from obesity to diabetes [61]. Lipotoxicity also plays an important role in β-cell destruction mediated by IL-1β production and secretion together with elevated glucose levels in cultured T2DM islets [62]. The neutralization of pro-inflammatory cytokines, especially IL-1β, may extinguish the inflammatory process of the pancreatic islets and thus normalize blood glucose levels and reduce insulin resistance [63]. High amounts of IL-1β may be the reason for insulin insensitivity in obese individuals. In the adipose tissue of such individuals, the expression of the NLRP3 inflammasome components, the activity of caspase-1, and the level of IL-1β are increased, all of which are directly correlated with insulin resistance, metabolic syndrome, and the severity of T2DM [64]. An important observation, however, is that not all obese subjects or patients prior to T2DM exhibit dyslipidemia. Overall, it must be underlined that IL-1β is only one of a few factors influencing the development of T2DM [65,66].

### 2.4. Interleukin 18

IL-18, which is also known as an interferon-gamma inducing factor, plays an important pro-inflammatory role, and it is involved in the induction of inflammatory agents as well as regulation of the cytotoxic activity of NK cells and T cells [67,68,69,70]. Moreover, IL-18 was found to assist the differentiation and activation of different T helper (Th) cells depending on the cytokine profile. Apart from its place in immune defense against infective pathogens, IL-18 participates in the pathogenesis of autoimmune and chronic inflammatory diseases [71].

The same two signals that are required for the production of IL-1β are also necessary to produce IL-18. The first signal is the synthesis of intracellular pro-interleukin, which occurs after stimulation of PRRs, such as TLRs situated in endosomes or on the cell surface and NLRs located in the cytoplasm, i.e., the ligation of PRR with its respected ligand [49]. In the case of IL-18, pro-IL-18, an inactive precursor of IL-18, is cleaved and converted by caspase-1 into the biologically active cytokine IL-18. After being processed, IL-18 is released into the extracellular space [72]. In 2012, Bellora et al. exposed that IL-18 is expressed not only as a secreted form but also as a membrane-bound form [73]. The inactive form has been observed in various cells, including dendritic cells, keratinocytes, macrophages, Kupffer cells, microglia, intestinal epithelial cells, astrocytes and osteoblasts. Summing up, many types of hematopoietic and nonhematopoietic cells have the potential to produce IL-18 [74,75].

The NLRP3 inflammasome is probably activated by a high glucose concentration or abnormal lipid metabolites, which accompany metabolic disorders. Subsequently, IL-18 and IL-1β are secreted, which can elicit inflammatory responses. IL-18 induces the production of TNFα, which in turn promotes the synthesis and release of IL-6 and CRP [76,77]. Since the discovery of IL-18 in 1995 by Okamura et al. [78], elevated levels of this cytokine have been associated with many health disorders and diseases, including obesity, metabolic syndrome, insulin resistance, diabetes, and atherosclerosis [79].

Krogh-Madsen et al. reported that TNF-induced insulin resistance is associated with an increased *IL18* gene expression in muscle tissue, suggesting that both TNF and IL-18 may play salient roles in pathogenesis of insulin resistance [80]. Lindegaard et al. identified in mice experiments that IL-18 can activate the adenosine monophosphate activated protein kinase (AMPK) signaling pathway, which enhances fat oxidation in skeletal muscles and sequentially reduces insulin resistance induced by HFD [81]. In this study, IL-18 receptor^−^/^−^ mice exhibited inflammation, weight gain, lipid deposition, and attenuated AMPK signaling in skeletal muscles, which indicated that IL-18 was involved in metabolic homeostasis, inflammation, and insulin resistance [81]. Esposito et al. reported that fasting IL-18 levels were higher in people with newly diagnosed T2DM than in matched healthy subjects. Moreover, a single high-fat meal was enough to significantly increase the levels of circulating IL-18 in subjects with and without T2DM [82]. Thorand et al. showed in a human population-based cohort study that the elevated serum levels of IL-18 were associated with a significantly increased risk of T2DM and that this association was independent of CRP and IL-6 [83]. Overall, these findings suggest that IL-18 induces inflammatory responses that may lead to insulin resistance and T2DM development. Recently, Zhuang et al. applied a Mendelian randomization method to study the causal relationship between IL-18 plasma levels and T2DM [84]. The authors concluded that the IL-18-associated T2DM risk is predominantly due to the role of pro-inflammatory cytokines in β-cell dysfunction.

## 3. Mechanism of Inflammasome NLRP3 Activation

Immune cells such as monocytes and macrophages activate the NLRP3 inflammasome in two-step process. The initial step clusters a process in which PAMPs, DAMPs, or environmental stress are recognized by TLRs or cytokines such as TNFα, leading to the activation of NF-κB, which evokes the expression and activation of NLRP3, pro-IL-1β, and pro-IL-18, and transcriptional protein modifications such as ASC phosphorylation, and NLRP3 de-ubiquitination. A secondary stimulus causes activation of the inflammasome by oligomerizing inactive NLRP3 [22,79,85]. Summing up, upon activation, NLRP3 undergoes a conformational change that exposes its nucleotide-binding domain (NBD) and N-terminal pyrin domain to allow for the formation of an oligomeric complex and facilitates the recruitment and activation of pro-caspase-1 [86].

Activation of the inflammasome has been explained using three different models favored in the literature: potassium ion (K^+^) efflux as induced by extracellular ATP (K^+^ channel model), the generation of reactive oxygen species (ROS) via PAMPs and DAMPs (ROS model), and crystalline structures causing lysosomal rupture-induced cathepsin B release (lysosomal damage model) [87,88]. The molecular mechanism by which these models activate the inflammasome remains unclear and has been the subject of much discussion by researchers [89].

The first model indicates that ion fluxes, especially K^+^ efflux, act as signals for NLRP3 inflammasome activation. Inhibition of K^+^ efflux by a high concentration of K^+^ in the cell culture can suppress NLRP3 inflammasome activation in response to most inflammasome activators [89,90]. This model is a crucial factor for the assembly and upregulation of NLRP3 complexes induced by the agonist ATP. The K^+^ efflux occurs through a pyrogenic P2X7-ATP-dependent pore that recruits a pannexin-1 hemichannel. This action permits extracellular NLRP3 agonists to enter the cytosol and to engage the NLRP3 protein complex, and triggers IL-1β secretion by the inflammasome [91]. In accord with this pattern, several studies have shown that an elevated concentration of extracellular potassium averts NLRP3 complex activation whereas a reduced cytoplasmic potassium concentration initiates the NLRP3 inflammasome activation [24]. Nevertheless, the molecular pathway between diminished levels of cytosolic potassium and NLRP3 activation requires further explanation [92].

The second model shows that the generation of ROS plays an essential role in the inflammasome activation. Almost all NLRP3 activators, including particulate matter, increase intracellular ROS production [93]. Furthermore, the inhibition of ROS with specific scavengers suppresses inflammasome activation in response to a range of NLRP3 activators [93]. Another research has revealed that thioredoxin-interacting protein can bind to NLRP3 in a ROS-dependent manner, additionally indicating the crucial role of ROS in inflammasome activation [94].

The third model revealed that cytosolic release of lysosomal cathepsin B consists in triggering inflammasome activation. In this type, phagocytosis of environmental molecules appears to activate the NLRP3 complex that shapes aggregates when absorbed by phagocytes. These crystalline structures trigger lysosomal effluent and release the essence into the cytosol through a mechanism mediated by lysosomal cysteine protease, cathepsin B, boosting the NLRP3 complex activation [85,95].

Furthermore, recent studies have suggested that posttranslational modifications of NLRP3 may be important in regulating its activation. NLRP3 is inactivated as a result of specific modifications, such as ubiquitination and phosphorylation, and it is activated after de-ubiquitination and de-phosphorylation [96,97].

## 4. Endothelial Dysfunction as the First Key Step to Activating the Inflammasome

Endothelial cells cover the inner surface of blood vessels in a single layer and perform several functions. They are responsible for the transport of nutrients (among them glucose), hormones, and macromolecules from the blood to the surrounding tissue, enabling its growth and proper metabolism [98]. Endothelial cells affect cell adhesion, the integrity of the vessel wall, vascular permeability, thrombus formation, and fibrinolysis; regulate blood flow; and maintain blood fluidity, leukocyte trafficking, angiogenesis and immunity [99,100]. They secrete mediators that regulate vascular tone—vasoconstrictors, i.e., endothelin-1 (ET-1) and thromboxane A2, and vasodilators, i.e., nitric oxide (NO), prostacycline, and endothelium-derived hyperpolarizing factor [101]. Endothelial cells ensure a barrier between the blood and tissues and therefore play a key role in the inflammatory response [102].

These cells constitute a line of defense against endogenous molecules or microbes causing infections or inflammation [103,104]. Many PAMPs and DAMPs can be detected by endothelial cells thanks to their innate immune system receptors [105,106]. Features of a damaged endothelium include phenotypic changes, inflammation, and impaired permeability, among others [103,107].

Impaired vasodilation, augmented pro-thrombotic and pro-inflammatory properties, and increased redox state all characterize endothelial dysfunction. In diabetes, endothelial dysfunction is defined as the early stage of different hyperglycemia-associated vascular diseases, such as atherosclerosis, that trigger vascular inflammation and ultimately atherosclerotic lesions. Endothelial dysfunction is developed at a very early stage of T2DM with a complex mechanism including altered cell signaling, increased oxidative stress, pro-inflammatory activation, and mitochondrial dysfunction [108,109]. This pathological condition in diabetes has been associated with high glucose and high fat levels in the blood, insulin resistance, and hypertension [110,111,112]. The expansion of hyperglycemia-induced vascular endothelium inflammation leads to endothelial barrier dysfunction, which eventually results in diabetes-associated vasculopathy [113]. Consequently, vascular complications consist of the primary cause of death and disability in patients with diabetes [114]. It has been noted that, in T2DM, insulin resistance and endothelial dysfunction occur before the development of overt hyperglycemia and that insulin resistance usually precedes hyperglycemia and diabetes by many years [100,115,116]. Many studies show that diabetes has been stipulated to have a close relationship with cardiovascular disease and that maintaining normoglycemia is key to preventing cardiovascular disorders [117,118,119].

Glucose transport to endothelial cells is insulin-independent and is mediated by glucose transporter 1 (GLUT1) in contrast to striated muscle cells, where it is mediated by the glucose transporter 4 (GLUT4). Therefore, the level of glucose in endothelial cells is the same as in the blood regardless of insulin sensitivity [120]. However, the insulin level in the blood affects the production of NO and ET-1 by the endothelium via two independent insulin-signaling pathways. Insulin resistance influences the production of NO but not the production of ET-1 [121]. Insulin-stimulated NO and ET-1 release are regulated by phosphatidylinositol 3-kinase (PI3K)-dependent and by MAPK-dependent signaling, respectively. Increased insulin signaling enhances the production of ET-1 [122,123,124,125]. Insulin resistance causes an imbalance between the production of NO and the secretion of ET-1. Selective impairment of PI3K activation and compensatory hyperinsulinemia has a direct effect on endothelial dysfunction through the pro-hypertensive, atherosclerotic, thrombogenic, and pro-coagulant effects of insulin [126].

**Figure 1:**
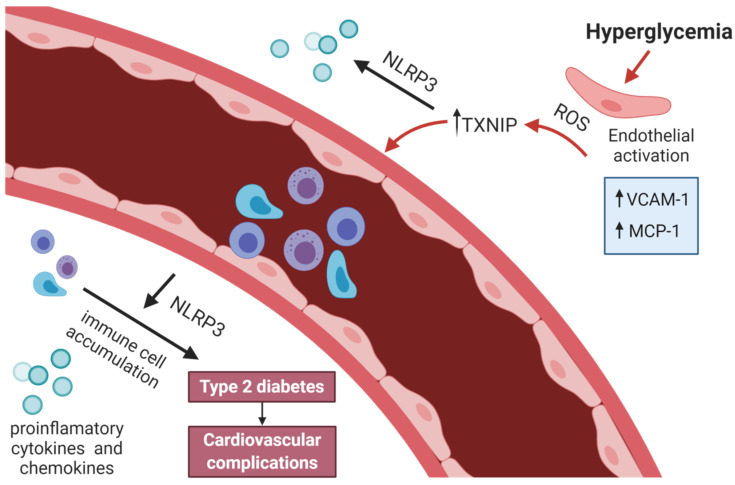
Links between NLRP3, endothelial dysfunction, and type 2 diabetes.

Hyperglycemia causes oxidative stress and endothelial dysfunction by the overproduction of ROS that causes cellular damage leading to diabetic complications through several biochemical pathways (Figure 1) [127,128].

The most important source of ROS is excessive activation of the mitochondrial electron transport chain, which is a major site of ATP production in mitochondria and glucose-induced activation of NAD(P)H-dependent oxidase [129,130,131,132]. The overproduction of ROS causes DNA damage. Poly (ADP ribose) polymerase 1 (PARP-1) is activated, and then, the glycolytic enzyme glyceraldehyde-3-phosphate dehydrogenase (G3PDH) is inhibited. This results in the accumulation of upstream glycolytic metabolites, which affect the polyol pathway, hexosamine pathway, and diacylglycerol (DAG) and protein kinase C (PKC) pathway, and in the generation of advanced glycation end products (AGEs) [133]. Increased polyol activity results in an accumulation of sorbitol and fructose and finally leads to the overproduction of H_2_O_2_ and ROS in general, an enhancement of oxidative stress, and the formation of methylglyoxal (precursor of AGEs) and DAG, which activate PKC [132,134,135]. PKC influences insulin receptor substrates 1 and 2, causing inactivation of the PI3K pathway and endothelial NO synthase (eNOS), and NO production in endothelial cells. Subsequently, the activity of NF-κB and the pro-oxidant enzyme NOX are increased [132,136]. Activation of NF-κB increases the production of vascular adhesion molecules, cytokines, and chemo-attractants and, consequently, the activation of inflammatory cells in the vascular wall [100]. AGEs are formed as a result of nonenzymatic glycation of proteins, nucleic acids, and lipids [137,138,139]. AGEs start to perform their functions as a result of binding to their receptors. Finally, AGEs induce inflammation characterized by the presence of pro-inflammatory cytokines and inflammatory molecules such as TNFα, IL-6, intercellular adhesion molecule 1 (ICAM-1), vascular cell adhesion molecule (VCAM-1), and MCP-1 (Figure 1) [140,141,142]. Activation of the hexosamine pathway leads to the dysregulation of O-linked glycosylation, which decreases PI3K/AKT signaling, which in turn reduces the phosphorylation of eNOS by serine/threonine protein kinase B (AKT). Eventually, the production of NO is decreased [143,144,145,146].

In insulin resistance states, the endoplasmic reticulum (ER) stress is activated and contributes to insulin resistance [147,148,149,150]. ER is the largest organelle in the cell and constitutes a dynamic structure that fulfills several functions including calcium storage, carbohydrate and lipid metabolism, protein synthesis, maturation, folding, and transport [151,152,153]. Excessive disrupted protein synthesis and accumulation of unfolded or misfolded proteins in the ER space results in ER stress, which leads to activation of a complex signaling network known as the unfolded protein response (UPR) [154]. The UPR is a pro-survival mechanism, of which the main role is to increase the protein folding capacity [150]. ER stress, similar to hyperglycemia, induces oxidative stress, causing excessive production of ROS and pathological effects [155,156]. Since there is a high demand for protein synthesis in diabetes, an overwhelming formation of nonnative disulfide bonds leads to overconsumption of glutathione (GSH). This is to scavenge ROS The depletion of stocks of reduced GSH in the cell leads to an increase in oxidative stress [157]. The misfolded proteins buildup inside the ER lumen, and large amounts of Ca^2+^ leak into the mitochondria, which enhances the production of ROS. Excessive Ca^2+^ inside the mitochondria leads to ROS production. Then, homeostasis of Ca^2+^ in the ER is lost, which stimulates ER stress and oxidative stress, causing endothelial dysfunction [132,158]. The contribution of ER stress to insulin resistance is through the UPR branch, protein kinase-like endoplasmic reticulum kinase (PERK) pathway [159].

The mechanistic target of rapamycin (mTOR) is a kinase that is a component of two different complexes: mTOR complex 1 (mTORC1) and mTOR complex 2 (mTORC2) [160,161]. The mTORC1 regulates cell growth and metabolism. Activation of mTORC1 affects protein and nucleotide synthesis, lipogenesis, and glycolysis; inhibits autophagy; and controls mitochondrial biogenesis and functions. In contrast, mTORC2 activation regulates cell survival and cytoskeletal organization and phosphorylates AGC kinases such as serum- and glucocorticoid-inducible kinase 1 (SGK1) and AKT. Both mTORC1 and mTORC2 are not active simultaneously—activation of mTORC1 deactivates mTORC2, and vice versa. The level of mTORC1 is increased in diabetes and metabolic stress conditions [162]. Chronic mTORC1 activation increases insulin resistance by regulating negative feedback through the phosphorylation of IRS-1 [163]. Previous studies have indicated an increase in mTORC1 activity in endothelial cells in response to glucose, insulin, oxidative stress, and angiotensin II. This demonstrates that mTORC1 may be an important mediator in several signaling pathways [164]. Nevertheless, the exact effect of mTORC1 on these pathways and, hence, on vascular function has not yet been characterized. The data reported by Decker and Pumiglia indicated that mTORC1 may be the critical determinant of eNOS phosphorylation. This finding provides new insight into eNOS uncoupling, endothelial dysfunction, and vascular disease accompanying diabetes [165]. Recently, Reho et al. demonstrated that mTORC1 signaling plays a key role in the regulation of vascular endothelial function by modulating ROS signaling [164]. Increased activity of mTORC1 causes elevated expression of pro-oxidative genes and the production of ROS, which may contribute to further dysfunction of endothelial cells. A blockade of the inhibitor of NF-κB subunit β activity with an anti-inflammatory, cell-permeable quinoxaline compound, BMS-345541, blocked the production of ROS due to increased mTORC1 activity. However, there is a little research into the role of mTOR signaling in the generation of endothelial dysfunction, which is responsible for inflammasome activation, and further studies are necessary.

Taking into consideration the role of NLRP3 inflammasome in the human body, suppressing the NLRP3 inflammasome could be a new approach to depleting hyperglycemic toxicity and to averting the onset of vascular complication [166].

Mice with Kawasaki disease, an inflammatory disease model, show impaired endothelium-dependent vasodilatation associated with increased caspase-1, IL-1β, and VCAM-1 expression [167]. Furthermore, NLRP3 deficiency protects endothelial function in hypercholesterolemic mice by depletion of vascular superoxide anion generation and increasing eNOS activity. In vitro experiments also indicate a modulatory role of NLRP3 on endothelial function since the silencing of the *NLRP3* gene prevents caspase-1 and IL-1β activation in endothelial cells stimulated with cell wall fragments of *Lactobacillus casei* [168]. Additionally, the expression of NLRP3 inflammasome constituents is increased in brain areas that control blood pressure in spontaneously hypertensive rats and is linked to extension vascular damage and high blood pressure. Chen et al. deliberated the role of the inflammasome activation mediating tight junction disruption, a critical event of endothelial impairment that leads to endothelial hyperpermeability in diabetes. The scientists observed that NLRP3 ablation prevented mice from tight junction impairment in the coronary arterial endothelium. Similarly, *NLRP3* gene silencing avoided high glucose-induced downregulation of tight junction proteins of mouse vascular endothelial cells in vitro [167]. They concluded that the ROS-dependent activation of endothelial NLRP3 inflammasome by hyperglycemia might be essential to initiate the mechanism provoking endothelial dysfunction and endothelial injury in diabetes [169].

## 5. Role of NLRP3 Inflammasome in Diabetes and Activation Inflammasome by Metabolic Signals

**Figure 2:**
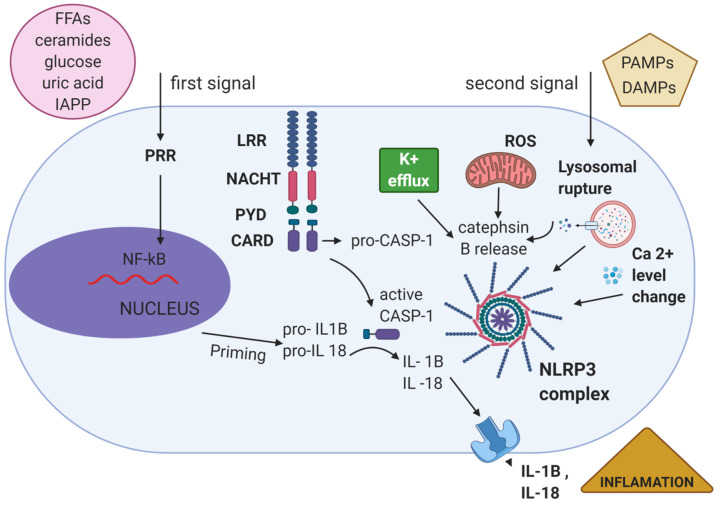
NLRP3 activation model.

The NLRP3 inflammasome seems to act as a sensor for metabolic danger signals (endogenous DAMPs and PAMPs) that accumulate during obesity, including saturated free fatty acids (FFAs), ceramides, high levels of glucose, uric acid, and Islet Amyloid Polipeptyde (IAAP) [27]. The NLRP3 inflammasome activation and successive IL-1β production have been first shown in pancreatic β-cells and islet-infiltrating macrophages [170]. This process results in IL-1β production, and numerous cytokines and chemokines are induced (Figure 2) [27].

Inflammation from nutrient overload and obesity is increasingly recognized in obesity-related diseases, including T2DM. Research confirms that caloric restriction and intermittent fasting reverse nutrient overload states in parallel with reducing inflammation [171]. Obesity-associated inflammation includes the activation of both the innate and adaptive immune systems. Moreover, obesity results in the activation of inflammatory cells in various lipid-accumulating organs, which in turn leads to the production of cytokines and acute-phase proteins [172]. The triggers for the pro-inflammatory pathways comprise the excess nutrient intermediates, including saturated fatty acids, ceramides, and excess glucose levels that function through pattern recognition receptors on leukocyte or adipocyte cell membranes [173]. The best studied organs/tissues for nutrient overload-related pathways activating immune response include the liver, pancreas, muscles, and adipocytes [174]. Collectively, human studies indicate that insulin resistance and obesity are strongly associated with increased NLRP3 expression in adipose tissue. Studies in obese mice corroborate this finding. Moreover, HFD increases NLRP3 expression in murine adipose tissue while calorie-restricted diet reduces its expression. Hence, NLRP3 blockade in mice protects against HFD-induced obesity and insulin resistance [175]. A variety of immune cells, including pro-inflammatory macrophages, affect adipose tissue homeostasis by increasing the production of cytokines such as TNF, IL-1β, and IL-6 [176]. NLRP3 inflammasome activation seems to be a key regulator of adipocyte differentiation and drives adipocytes towards more insulin resistance. Consistently, caloric restriction and exercise-mediated weight loss in obese subjects with T2DM reduce *NLRP3* (also known as *CIAS1*) and *IL1B* gene expressions in abdominal subcutaneous adipose tissue, improving insulin sensitivity [177]. Esser et al. demonstrated an increased expression of *NLRP3* and *IL1B* in visceral adipose tissue from metabolically unhealthy obese patients compared with metabolically healthy obese patients. These authors also reported that *NLRP3* expression positively correlated with insulin resistance [178]. Yin et al. found that *NLRP3* expression was increased in subcutaneous adipose tissue from mice fed for 3 months with a HFD compared with control mice [179]. Wang et al. showed that *NLRP3* and *Caspase-1* expressions were increased in epididymal fat from db/db mice compared with wild-type mice [180]. Finucane et al. demonstrated that *NLRP3*, *Caspase-1*, and *IL1B* expressions in adipose tissue were higher in mice treated for 6 months with a saturated fatty acid HFD in comparison with mice fed with a monounsaturated fatty acid HFD [181]. In the same study, it was demonstrated in vitro that the latter diet can reduce adipose IL-1β secretion and insulin resistance. The authors further showed that the effect of the monounsaturated fatty acid is related to the ability to block ATP-induced processing of IL-1β in an AMPK-dependent manner.

The NLRP3 inflammasome is extensively studied in inflammation prognosis and progression of T2DM. Liu et al. reviewed the crucial role of NLRP3 in the pathogenesis of T2DM. It has been demonstrated that IL-1β is elevated in T2DM patients, suggesting that IL-1β may be linked with the development of T2DM [182]. Excessive IL-1β production in T2DM has a few consequences: (1) it induces the expression of other inflammatory mediators (IL-18, IL-33) using IL-1 receptor signaling that amplifies inflammatory reaction [183]; (2) it evokes oxidative stress as well as ER stress, which are both closely linked to T2DM [184,185]; and (3) it activates c-Jun N-terminal kinases (JNKs), inducing serine phosphorylation of insulin receptor substrate 1 (IRS-1) and attenuating the activity of the insulin-PI3K/AKT signaling pathway in insulin-sensitive tissues [186]. Bitto et al. exposed that NLRP3, ASC, caspase-1, IL-18, and IL-1β are upregulated during wound healing in animal models of T2DM in comparison with healthy animals [187]. Kim et al. demonstrated that NLRP3 can be suppressed by γ-tocotrienol, delaying the progression of T2DM [188]. Coll et al. reported that MCC950, which inhibits the NLRP3 inflammasome, can be applied as a potential anti-inflammatory therapy in T2DM [189]. More evidence for a pathogenic role of NLRP3 inflammasome in T2DM comes from a study reporting that glyburide suppresses inflammasome-mediated IL-1β release in monocytes [190]. Metformin was also reported to decrease IL-1β levels, which was attributed to activating AMPK [191]. Henriksbo et al. revealed that Fluvastatin provokes inflammation and insulin resistance in adipose tissue via the upregulation of NLRP3, which is consistent with the increased expression of NLRP3 in inflamed adipose tissues of T2DM patients [192]. These observations confirm that the activation of NLRP3 inflammasome is related to insulin resistance. Furthermore, wound healing is disrupted in T2DM patients [193]. The above data clearly show that much research was conducted to explain the association of NLRP3 inflammasome and T2DM. Nevertheless, the mechanism of NLRP3 inflammasome activation in T2DM is not fully understood and requires further studies [194].

The role of NLRP3 inflammasome in metabolic syndrome and T2DM can be split into two subcategories. The first subcategory contains mediated roles by sensing endogenous inflammasome activators, whereas the second contains indirect roles related to inflammasome-associated alteration, leading to manipulation of the gut microbiota. It is worth stressing that T2DM has been the first metabolic disease shown to involve NLRP3 [195,196].

Elevated plasma FFAs, mainly a result of increased high fat diet consumption, contributes to the development of T2DM [197]. Recent studies revealed an association between NLRP3 inflammasomes and metabolic disease-related lipid species, such as saturated fatty acids (SFAs) and ceramides (Figure 2) [198]. An analysis from the Ting laboratory demonstrated that the fatty acid palmitate in human plasma can activate the NLRP3 inflammasome [199]. Kien et al. uncovered that lowering the dietary palmitate to anoleate ratio diminishes cytokines in leukocytes and reduces redox-sensitive gene expression in muscles [200]. Palmitic acid is one abundant FFA that activates the NLRP3 inflammasome via mitochondrial ROS production and lysosomal destabilization, thus promoting insulin resistance. Like palmitate, the unsaturated fatty acid oleate, is likewise highly abundant in plasma; however, in contrast to palmitate, oleate was shown not to activate the NLRP3 inflammasome [199,201]. Interestingly, whereas saturated fatty acid activates the NLRP3 inflammasome in mice and contributes to insulin resistance, unsaturated fatty acid attenuates IL-1β-mediated insulin resistance by preserving AMPK activity [202]. Several studies highlight that dietary fatty acid composition is sensed by the NLRP3 inflammasome that modulates its response depending on fat type. Different cell types respond to FFAs and assemble inflammasome components, inducing IL-1β release [181].

Palmitate-induced NLRP3 inflammasome complex formation disrupts endothelial tight junctions, leading to the onset of endothelial injury during obesity. Intestinal epithelial cells react to a high-cholesterol diet by activating caspase-1 after the IL-1β-dependent accumulation of myeloid cells in the intestine. In monocytes, palmitate activates caspase-4/5 and prompts the release of IL-1β and IL-18 [203,204,205].

Increased levels of SFAs promote the synthesis of ceramides. This lipid species production is associated with an inflammatory response during obesity-induced diabetes. Ceramide has been shown to evoke NLRP3 inflammasome activation in cultured macrophages and in adipose tissue explants of diet-induced obese mice, which, upon exposure to this lipid, stimulate NLRP3-dependent caspase-1 activation [177,206].

Hyperglycemia is one of the hallmarks of T2DM [207]. It has been known for almost two decades that the NLRP3 inflammasome is activated in response to high levels of glucose (Figure 2) [182,208]. Some reports show that β cells in pancreatic islets produce IL-1β in response to high glucose, which contributes to glucotoxicity and results in functional impairment and apoptosis of β cells [58]. Aside from β cells, hyperglycemia is also reported to increase IL-1β generation in adipose tissue and a cardiomyocyte cell lines [209]. Recent evidence has suggested that high glucose levels promote *IL1B* mRNA transcription [210]. Glucose has been shown to activate the protein kinase C alpha (PKCα), and via phosphorylation of p38, MAPK and extracellular signal-regulated kinases 1/2 (ERK1/2) lead to NF-κB activation and subsequent *IL1B* transcription in monocytes, hence priming cells for inflammasome activation [211]. High glucose concentration may supply the priming signal for transcription of *IL1B* by activation of thioredoxin-interacting protein (TXNIP), which afterwards facilitates boosted IL-1β expression levels. Additionally, high levels of glucose promote the production of ROS, and that is sufficient as the second signal that promotes inflammasome activation of caspase-1 and processing of IL-1β in pancreatic islets [212,213]. TXNIP, also known as thioredoxin-binding protein 2, is a protein that interacts and negatively regulates the expression and function of thioredoxin (TXN). TXNIP acts as a crucial regulator of lipid and glucose metabolism through pleiotropic actions including regulation of β cell function, peripheral glucose uptake, adipogenesis, hepatic glucose production, and substrate utilization. The upregulation of TXNIP in animal models has been shown to induce apoptosis of pancreatic β cells and to reduce insulin sensitivity in peripheral tissues like skeletal muscles and adipose tissue. On the contrary, TXNIP-deficient animals are protected from diet-induced insulin resistance and T2DM [213,214]. Alhawiti et al. indicated that TXNIP levels are elevated in people with T2DM and that its expression is strongly induced by glucose [215]. It seems to be interesting that, upon activation, TXNIP is able to directly interact with NLRP3 in a ROS-dependent manner, leading to the activation of caspase-1 and the processing of IL-1β in pancreatic islets. However, these observations could not be reproduced in bone-marrow-derived macrophages lacking TXNIP and exhibited in high glucose levels [216].

Obesity is accompanied by an increased level of uric acid. Uric acid can form crystals as NLRP3 activators [217]. Uric acid is a product of purine catabolism released from ischemic tissues and dying cells. After crystallization, uric acid activates the immune system by reducing NO availability, increasing ROS production, stimulating chemotaxis, and activating the NF-κB and MAPK pathways [218]. Uric acid crystals also induce the release of pro-inflammatory cytokines such as IL-1β, secreted during inflammasome formation [219].

In 2010, Masters et al. demonstrated that amylin, known also as islet amyloid polypeptide (IAPP), which is secreted by pancreatic β cells besides insulin, can be converted from a soluble to amyloid form and can activate the NLRP3 inflammasome in mouse macrophages (Figure 2) [220]. Human IAPP peptides have the tendency to misfold and to form insoluble aggregates [221]. IAPP production is chronically stimulated in obese individuals due to high blood glucose levels, and amyloid IAPP accumulates in the pancreas. Similar to many other particulate substances, IAPP aggregates are potent NLRP3 activators [222]. In a recent study, the stimulation of macrophages with human IAPP was shown to induce the cleavage of caspase-1 and the production of IL-1β in an NLRP3-dependent manner [223].

Intestinal microflora are involved in metabolic, immunological, and protective functions [224]. A change in the composition of the intestinal microbiota, i.e., a process called dysbiosis, has been reported to play a key role in the pathogenesis of inflammatory diseases such as T2DM [225]. Inflammasomes play an important role in regulating the composition of the gut microflora, as highlighted by recent studies in mouse models [226]. In this study, a susceptibility to colitis and tumorigenesis was indicated as the result of the absence of components of the inflammasome associated with dysbiosis [226]. Accumulating evidence strongly suggests that the inflammasome structure is responsible for the epithelium intestinal integrity and protection against pathogenic attack [166]. The mechanisms and factors involved in activation of the inflammasome by intestinal microbiota are unknown. However, it has been reported that some species of pathogenic bacteria such as *Citrobacterrodentium*, *Listeria monocytogenes*, *Clostridium difficile*, and *Salmonella typhimurium* growing in the intestine appear to be associated with the deficiency of IL-1β, IL-18, and caspase-1 [227,228].

## 6. Epigenetic Regulation of NLRP3 Inflammasome by microRNA

MicroRNAs (miRNAs) are small, endogenous noncoding RNAs that are 19–24 nucleotides (nt) in length and exert regulatory functions through complementary base pairing to the 39 untranslated regions (39 UTRs) of protein-coding messenger RNAs (mRNAs). The discovery of miRNAs disclosed a new dimension in the posttranscriptional regulation of gene expression. Humans have more than 2500 miRNAs that participate in most cellular processes [229,230,231]. The expression of miRNA is deregulated during diseases such as cancer, influenza, leukemia, and T2DM [232,233]. There have been a few dozens of miRNAs reported to be involved in either regulating the NLRP3 inflammasome or developing inflammation, endothelial dysfunction, or T2DM [234,235,236]. A review of all these miRNAs is beyond the scope of this manuscript. However, below, we present those miRNAs that are altered in T2DM and are also known to be involved in either NLRP3 inflammasome activation or endothelial dysfunction. We start with miRNA-223, which is a key regulator of NLRP3 activity. The role of miRNAs in controlling innate immune responses has primarily been aimed at TLR signal-transduction pathways. Respective miRNAs were identified to be induced upon TLR activation targeting mRNAs encoding elements of the TLR-signaling system itself. These regulatory systems have evolved to allow a strong initial immune response that is gradually dampened down after secondary induction of the regulating miRNAs [237,238].

miRNA-223 is a highly conserved miRNA. The gene encoding miRNA-223 is located within the q12 locus of the X chromosome [239,240]. It is specifically expressed in myeloid lineage and triggers myeloid differentiation of progenitor cells to maintain granulocyte function [241]. These molecular particles are involved in the regulation of hematopoiesis, immune response, and different types of inflammation disorders. A few studies have indicated that miRNA-223 is deregulated in T2DM [242,243]. This suggests that T2DM is an inflammatory disease with pathogenesis driven at least in part by pro-inflammatory cytokines. Quantitative miRNA expression analyses revealed that these particles were consistently upregulated in hearts of the insulin-resistant patients with T2DM. This effect was connected with the miRNA-223 role in GLUT4 regulation and glucose metabolism. In vitro, the overexpression of miRNA-223 in cardiomyocytes increased the total GLUT4 level and induced GLUT4 translocation from the cytoplasmic compartment to the cell membrane. In vivo, the inhibition of miRNA-223 in the heart resulted in a significant decrease in GLUT4 expression [244]. In individuals with T2DM, the increased miRNA-223 and GLUT4-dependent glucose uptake may be insignificant in the periphery, but in the heart, this may have a marked effect and may increase metabolic processes that could be detrimental. This possibility is suggested by the fact that myocardial infarction is the primary cause of mortality in people with T2DM [245,246].

The miRNA-223 is a crucial regulator of NLRP3 inflammasome activity. Tight transcriptional regulation trough miRNA-223 controls inflammasome activation by manipulating mRNA levels of NLRP3 [247]. The inhibition of NLRP3 expression by miRNA-223 occurs through a conserved binding site within the 3′UTR of NLRP3, leading to limited NLRP3 inflammasome activity. Haneklaus et al. proved that miRNA-223 targets the 3′UTR of NLRP3 and that miRNA-223 expression decreases as monocytes differentiate into macrophages and NLRP3 protein levels increase [246]. The overexpression of miRNA-223 averts the accumulation of NLRP3 protein and inhibits IL-1β generation by the inflammasome [243]. Further investigations are needed to elucidate the functional role and therapeutic potential of miRNA-223 in metabolic disorders. This may provide new insights into the epigenetic regulation of NLRP3 inflammasome [248,249].

Up until now, there were four miRNAs reported to play regulatory roles in T2DM as well as endothelial dysfunction and NLRP3 inflammasome, i.e., miRNA-9, miRNA-21, miRNA-133, and miRNA-146a [234,235,236].

The gene encoding miRNA-9 is located on human chromosome 1 (1q22) and is highly expressed in the brain area. miRNA-9 is linked to the secretion of insulin [250,251]. The relation between β-cell function and miRNA-9 was found through in vitro and in vivo experiments. Ramachandran et al. reported that miRNA-9 targets and regulates Sirt1 expression in insulin-secreting cells. This targeting is relevant in pancreatic islets, where decreasing Sirt1 protein levels are accompanied by high miRNA-9 expression during glucose-dependent insulin secretion. This functional interplay between insulin secretion, miRNA-9, and Sirt1 expression could be relevant in diabetes [252]. In 2006, Plaisance et al. demonstrated that insulin secretion can also be affected by the overexpression of miRNA-9, which targets the transcription factor Onecut-2 by reducing its amount, leading to the increased expression of Granuphilin/Slp4, a negative regulator of insulin secretion [253].

The *MIR21* gene encoding miRNA-21 is located on human chromosome 17 (17q23.1) [254]. These molecules are highly expressed in endothelial cells, cardiomyocytes, and cardiac fibroblasts, acting on a variety of targets related to apoptosis and inflammation. Both in vitro and in vivo studies showed that miRNA-21 was upregulated in T2DM and obesity, suggesting a potential role in common aspects of the disease pathogenesis [255]. miRNA-21 is involved in the vascular endothelial growth factor (VEGF) and transforming growth factor beta (TGF-β) signaling pathways and has been shown to be a target for weight reduction in vivo [256].

Diabetic condition in vivo as well as the persistent exposure to high insulin and glucose in vitro seem to contribute to the upregulation of miRNA-21 in adipocytes. This miRNA was reported to be upregulated in the kidneys of diabetic mice and in mesangial cell lines grown in high-glucose conditions [257]. At the molecular level, in diabetic conditions, miRNA-21 was reported to increase the downstream AKT/target of rapamycin complex 1 (AKT/TORC1) activity and to increase NF-κB signaling pathways [258]. Ling et al. demonstrated that miRNA-21 reversed high-glucose and high insulin-induced insulin resistance in 3T3-L1 adipocytes. Overexpressing miRNA-21 extensively improved the insulin-induced phosphorylation in AKT, glycogen synthase kinase 3 beta (GSK3β), and GLUT4 in insulin-resistive adipocytes [259]. Zhang et al. showed that miRNA-21 deficiency increased the production of ROS and induced endothelial dysfunction. Endothelial miRNA-21 may play a critical role in vascular remodeling by regulating transforming growth factor beta 1 (TGF-β1) signaling [260].

miRNA-133, which is coded by three genes (*MIR133A1*, *MIR133A2*, and *MIR133B* located on chromosomes 18, 20, and 6, respectively), is mainly expressed in the muscles and controls the pathogenesis of insulin resistance [261]. Bandyopadhyay et al. reported that overexpression of miRNA-133a-1-enhanced secretion of IL-1β and the activation of caspase-1 resulting from upregulation of the NLRP3 inflammasome [262]. Contrary to miRNA-133a-1, miRNA-133b inhibits NLRP3 inflammasome. It binds to the 3′UTR of NLRP3 and downregulates its activation, which leads to the decreased expression of caspase-1, ASC, IL-18, and IL-1 [263].

miRNA-133, miRNA-223, as well as miRNA-1 are expressed in a diabetic heart and take part in the development of diabetic cardiomyopathy. miRNA-133 promotes the development of diabetic cardiomyopathy by inhibiting the expression of GLUT4 or Kruppel-Like Factor 15, which reduce glucose uptake and metabolism in cardiomyocytes [264].

A few studies support the notion that miRNA-146a, that is coded by *MIR146A* located on human chromosome 5 (5q33.3), has anti-inflammatory effects, and miRNA-146a deficiency enhances inflammatory responses, promoting increased activation of the NLRP3 inflammasome and the secretion of IL-1β and IL-18 [234,265]. miRNA-146a directly downregulates the production of pro-inflammatory cytokines by targeting tumor necrosis factor receptor (TNFR)-associated factor 6 (TRAF6) and IL-1 receptor associated kinase (IRAK1) [266,267]. In mice with miRNA-146a deficiency, the production of pro-inflammatory cytokines IL-1β and TNFα is increased, and the genes of NRLP3, ASC, and caspase-1 are remarkably overexpressed [268]. Cowan et al. reported that prolonged exposure of saturated fatty acids to the β-cell line MIN6 cells and pancreatic islets increased the expression of miRNA-146 [269].

Other miRNAs with potential roles in the regulation of the inflammasome complex and associated with the development of diabetes include miRNA-7, miRNA-132, and miRNA-148 [234,235].

The gene encoding miRNA-7 is located on human chromosome 9 (9q21.32) and directly regulated by the transcription factor HoxD10 [270]. miRNA-7 is associated with insulin secretion, β-cell development, and glucose homeostasis. The molecules are highly expressed in islet cells and regulate components necessary for insulin exocytosis [271]. Recent studies pointed out the higher expression level of miRNA-7 in human pancreatic fetal and adult endocrine cells. These studies revealed that the inhibition of miRNA-7 during the embryonic stage results in general downregulation of insulin production, in a decrease in β-cell count, and in glucose intolerance in the postnatal period [272,273].

Upregulation of miRNA-132 may be associated with prolonged hyperglycemia. It was observed in vitro in islets exposed to high-glucose concentrations and in vivo in rat islets in a non-obesity T2DM model [274]. Bijirk et al. reported the in vivo effect of cholesterol-conjugated antagomirs targeting miRNA-132 on islet function in mice [275]. They verified that miRNA-132 expression was decreased in pancreatic islets and that treatment resulted in increased insulin secretion and a reduction in blood glucose levels [275]. Nesca et al. investigated that the expression of miRNA-132 increased as a result of incubation with palmitate while the expression of other miRNAs decreased after the same treatment [276].

miRNA-148 was found to be overexpressed in adipose tissue of high fat-fed mice and obese human subjects, promoting adipogenesis in mesenchymal stem cells by targeting proto-oncogene protein Wnt1. miRNA-148 downregulates insulin promoter activity and insulin mRNA levels [277].

Several other miRNAs are involved in the development and progression of endothelial dysfunction and T2DM. They are miRNA-29b, miRNA-122, miRNA-126, and miRNA-143 [235,236].

miRNA-29b is encoded by a gene located on human chromosome 7 (7q32.3). Studies have shown that miRNA-29b expression is increased in the liver, muscle, and fat of diabetic rats, and a relationship with insulin resistance has been shown in cellular experiments [278]. miRNA-29b mainly mediates the insulin pathway by inhibiting proteins that enhance insulin signaling. In addition, it can prevent insulin secretion by inhibiting monocarboxylate transporter 1 (MCT1) [279]. miRNA-29b has also been shown to increase blood pressure and endothelial inflammation [280].

The gene encoding miRNA-122, located on human chromosome 18 (18q21.31) miRNA-122 is mainly expressed in the liver, and it regulates the expression of various genes associated with cholesterol and fatty acid metabolism. In a population-based study, it was shown that circulating levels of miRNA-122 were elevated in people with metabolic syndrome or T2DM. Moreover, they were also associated with the risk of new-onset metabolic syndrome and T2DM in the general population [281]. miRNA-122 levels have been higher in the sera from hyperglycemic patients with acute coronary syndrome compared to sera from normoglycemic patients suffering from acute coronary syndrome [282]. Young et al. demonstrated that miRNA-122 contributed to the endothelial dysfunction in hypertension by reducing L-arginine and NO metabolism [283]. Recently, Wu et al. reported that the inhibition of miRNA-122 reduced atherosclerotic lesion formation in ApoE¯/¯ mice by regulating neuronal Per-Arnt-Sim domain protein 3 (NPAS3)-mediated endothelial to mesenchymal transition [284].

The *MIR126* gene is located on human chromosome 9 (9q34.3). In 2010, Zampetaki et al. reported for the first time that endothelial miRNA-126 levels were deregulated in people with T2DM, which suggested a potential role of this miRNA as a novel biomarker for a newly diagnosed T2DM [243]. This was then confirmed by other authors [285]. Moreover, in an in vitro study, it was revealed that hyperglycemia reduced the miRNA-126 content of endothelial apoptotic bodies and affected miRNA-126 release from endothelial cells, suggesting that this miRNA can be a potential soluble biomarker for endothelial dysfunction or atherosclerosis [243]. miRNA-126 is considered the main regulator of endothelial homeostasis and vascular integrity [286]. Harris et al. stated the involvement of miRNAs in endothelial cells activation and dysfunction [287]. They demonstrated that VCAM-1 is a potential target of miRNA-126. Endogenous miRNA-126 decreases VCAM-1 expression, thereby suppressing leukocyte interactions with endothelial cells. The above data suggest that miRNA-126 can regulate vascular inflammation [287]. miRNA-126 can promote insulin resistance by inhibiting IRS1 [288].

Xu et al. documented a correlation between upregulated miRNA-143, located on human chromosome 5 (5q32); endothelial dysfunction; and atherosclerotic plaque formation. Low miRNA-143 levels were accompanied by the elevated glycolysis rate in endothelial cells. Contrarily, the overexpression of miR-143 suppressed glycolysis through direct targeting of hexokinase 2 [289]. Interestingly, miRNA-143 was upregulated in atherosclerotic plaque samples in comparison with normal arteries. Jordan et al. used two obesity mouse models, i.e., db/db mice and diet-induced obesity mice, to demonstrate increased hepatic miRNA-143 expression. According to this study, the overexpression of miRNA-143 may reduce insulin sensitivity by downregulating oxysterol-binding-protein-related protein 8 (ORP8) and by impairing insulin-induced AKT activation and glucose homeostasis [290].

## 7. Conclusions

This review summarizes the links between the NLRP3 inflammasome, endothelial dysfunction, and type 2 diabetes. The role of NLRP3 as an indicator of metabolic disturbance has been clearly demonstrated. Inflammasome activity is crucial for host responses to microbial pathogens and cytokine production by the innate immune system shaping the adaptive immune response. Understanding the balance between favorable and noxious inflammasome activation is necessary in order to shape this activation for the benefit of the patient. Endothelial cells become active participants and regulators of the inflammatory response in the event of inflammation or infection as they are among the first cells to come into contact with endogenous particles or microbes Taking into consideration the role of NLRP3 inflammasome in the human body, suppressing the NLRP3 inflammasome could be a new approach in depletion of hyperglycemic toxicity and averting the onset of vascular complication in people with T2DM.

We revealed that the advancement of research increased the understanding of potential mechanisms that affect endothelial function through the NLRP3 inflammasome activation pathway. A better understanding of the interactions between endothelial dysfunction and the NLRP3 inflammasome-regulated pathways may open up a new avenue for effective treatment of cardiovascular complications in metabolic diseases including T2DM.

In this review, we also summarized previous studies on the molecular mechanisms of inflammasome activation. Discoveries in fundamental and preclinical research in recent years have demonstrated great potential for miRNA in the treatment of diseases linked to NLRP3 dysfunction. A few miRNAs are deregulated in T2DM, which is compatible with an emerging body of evidence suggesting that this is an inflammatory disease with pathogenesis driven at least in part by pro-inflammatory cytokines. Thanks to the increase in molecular information about inflammasomes, new therapeutic options are expected for patients with inflammatory diseases. This may offer potential novel therapeutic perspectives in T2DM prevention and treatment.

Finally, it must be stressed that inflammasomes other than NLRP3, such as AIM2, NLRP1, NLRC4, and NLRP6, have not been thoroughly investigated so far in the context of the development of endothelial dysfunction and the impact on pathogenesis and progression of T2DM.

## Data Availability

No new data were created or analyzed in this study. Data sharing is not applicable to this article.

## Abbreviations

3′UTR	3′untranslated region
39 UTRs	39 untranslated regions
AGEs	advanced glycation end products
AKT	serine/threonine protein kinase B
AIM	absentin-melanoma
ALRs	absentin-melanoma-like receptors
AMPK	adenosine monophosphate-activated protein kinase
ASC	apoptosis-associated speck-like protein
CARD	C-terminal caspase recruitment domain
CRP	C-reactice protein
CTLs	C-type lectins
DAG	diacylglycerol
DAMPs	damage-associated molecular patterns
dsDNA	bacterial double-stranded DNA
ET-1	endothelin-1
ER	endoplasmic reticulum
ERK1/2	extracellular signal-regulated kinases 1/2
eNOS	endothelial NO synthase
FFAs	saturated free fatty acids
G3PDH	glyceraldehyde-3-phosphate dehydrogenase
GSH	glutathione
GSK3β	glycogen synthase kinase 3 beta
GLUT1	glucose transporter 1
GLUT4	glucose transporter 4
HFD	high-fat diet
IAAP	islet amyloid polipeptyde
ICAM-1	intercellular adhesion molecule 1
ICE	interleukin-1β-converting enzyme
IDF	International Diabetes Federation
IL	interleukins
IRAK1	IL-1 receptor-associated kinase
IRS-1	insulin receptor substrate-1
JNKs	c-Jun N-terminal kinases
LRR	leucine-rich repeats
MAPK	mitogen-activated protein kinases
MCP1	monocyte chemoattractant protein 1
MCT1	monocarboxylate transporter 1
miRNAs	microRNAs
mRNAs	messenger RNAs
mTOR	mechanistic target of rapamycin
mTORC	mechanistic target of rapamycin complex
NBD	nucleotide-binding domain
NF-κB	nuclear factor kappa-light-chain-enhancer of activated B cells
NLRP	nucleotide-binding oligomerization domain-like receptor with a pyrin domain
NLRP3	protein 3 inflammasome complex
NLR	Nod-like receptor
NO	nitric oxide
NOD	nucleotide-binding oligomerization domain
NOD	nucleotide oligomerization domain
NPAS3	neuronal Per-Arnt-Sim domain protein 3
NT	nucleotides
ORP8	oxysterol-binding-protein-related protein 8
PAMPs	pathogen-associated molecular patterns
PARP-1	poly (ADP ribose) polymerase 1
PERK	protein kinase-like endoplasmic reticulum kinase
PI3K	phosphatidylinositol 3-kinase
PKC	protein kinase C
PKCα	protein kinase C alpha
PRRs	pattern recognition receptor
PYD	pyrin domain
RIG-I	retinoic acid inducible gene-I
RLRs	RIG-I-like receptors
ROS	reactive oxygen species
SFAs	saturated fatty acids
SGK1	serum- and glucocorticoid-inducible kinase 1
T2DM	type 2 diabetes mellitus
TGF-β	transforming growth factor beta
Th	T helper
TLRs	toll-like receptors
TNFα	tumor necrosis factor alpha
TNFR	tumor necrosis factor receptor
TORC1	target of rapamycin complex 1
TRAF6	TNFR-associated factor 6
TXN	thioredoxin
TXNIP	thioredoxin-interacting protein
UPR	unfolded protein response
VCAM-1	vascular cell adhesion molecule
VEGF	vascular endothelial growth factor
